# Evaluation of cytology versus human papillomavirus-based cervical cancer screening algorithms in Bhutan

**DOI:** 10.18632/oncotarget.19783

**Published:** 2017-08-02

**Authors:** Ugyen Tshomo, Silvia Franceschi, Tshokey Tshokey, Tashi Tobgay, Iacopo Baussano, Vanessa Tenet, Daniëlle A.M. Heideman, Peter J.F. Snijders, Gary M. Clifford

**Affiliations:** ^1^ Department of Obstetrics & Gynaecology, Jigme Dorji Wangchuck National Referral Hospital, Thimphu, Bhutan; ^2^ International Agency for Research on Cancer, Lyon, France; ^3^ Department of Pathology & Laboratory Medicine, Jigme Dorji Wangchuck National Referral Hospital, Thimphu, Bhutan; ^4^ Department of Pathology, VU University Medical Center, Amsterdam, The Netherlands

**Keywords:** cytology, methylation, human papillomavirus, cervical cancer, Bhutan

## Abstract

To evaluate the performance of existing versus alternative cervical cancer screening protocols in Bhutan, cervical exfoliated cells were collected for cytology and high-risk human papillomavirus (HR-HPV) testing among 1,048 women aged 30-69 years. Conventional smears were prepared and read locally. HR-HPV was tested by GP5+/6+ polymerase chain reaction, followed by genotyping and human DNA methylation analysis among HR-HPV-positives, in Europe. Test positivity was 7.5% for ASCUS or worse (ASCUS+) cytology and 14.0% for HR-HPV. All women with ASCUS+ and/or HR-HPV positivity (n=192) were recalled for colposcopy, among whom a total of 29 cases of histologically confirmed cervical intraepithelial neoplasia grade 2 or worse (CIN2+) were identified. An additional 7 CIN2+ cases were imputed among women without colposcopy. Corrected sensitivities for CIN2+ and CIN3+ were 61% and 74% for ASCUS+, 86% and 96% for HR-HPV, and 47% and 70% for ASCUS+ triage of HR-HPV. Specificity varied from 88% for HR-HPV up to 98% for ASCUS+ triage of HR-HPV, similarly for CIN2+ and CIN3+. Among HR-HPV-positive women with biopsies, methylation analysis offered similar discrimination of CIN2/3 and cervical cancer as ASCUS+, and better than HPV16/18 genotyping alone, but sample sizes were limited. In conclusion, the performance of cytology in Bhutan is in the mid-range of that reported in other screening settings. HR-HPV testing has the potential to improve detection of CIN2+, albeit with a higher referral rate for colposcopy. Cytological triage of HR-HPV-positives (performed in the absence of knowledge of HR-HPV status) reduced referral but missed more than one third of CIN2+.

## INTRODUCTION

Cervical cancer represents the most common cancer among females in Bhutan [1], where a national cytology-based screening program exists since 2000 [2]. The program is provided free of charge and recommends Papanicolaou (Pap) smears every three years for women aged 25-60 years, followed by colposcopy for screen-positive women. Due to limitations in trained personnel, most Pap smears are read, and most colposcopies are performed, in only two regional centres, the capital Thimphu, and Mongar in Eastern Bhutan. Although campaigns are also conducted in more remote, rural areas, the majority of cytology and work-up of screen-positive women is provided in national referral hospitals, so that the population coverage of at least one lifetime Pap smear has been estimated to vary between 20% and 60% according to district [3, 4]. More recently, there have been attempts to introduce cervical screening using self-collection of samples for the detection of high-risk human papillomavirus (HR-HPV) [5].

Indeed, during the last decade, cervical cancer screening has shifted towards the molecular detection of HR-HPV, the main cause of cervical cancer, allowing for increased automation of diagnostic procedures. Randomized trials in high-income countries among regularly screened women show that HR-HPV testing provides 60–70% greater protection against invasive cervical carcinomas over cytology, and allows extension of screening intervals [6]. Large studies conducted in low and middle income countries (LMICs) have also shown good cross-sectional [7–15] and prospective [16, 17] accuracy of HR-HPV testing versus cytology in largely unscreened populations. With respect to triage of HR-HPV-positive women, atypical squamous cells of undetermined significance (ASCUS) cytology or worse (ASCUS+), alone or in combination with HPV16/18 genotyping [18–20], is the recommended approach in high-income countries (HIC), but host gene methylation [21–23] offers an alternative molecular triage option.

Within the framework of a collaboration between the Ministry of Health (MoH) of Bhutan and International Agency for Research on Cancer (IARC) [3, 24], we here report the performance of a cervical screening program carried out in Thimphu, Bhutan, among women aged 30 years or older. The cross-sectional performance of Pap smear and HR-HPV using a clinically validated test for cervical screening (GP5+/6+) [25], plus the potential use of HPV16/18 genotyping and DNA methylation markers to triage HR-HPV-positives, were evaluated based upon a gold standard of colposcopy and histologically proven cervical intraepithelial neoplasia (CIN) grade 2 or worse (CIN2+) and CIN grade 3 or worse (CIN3+).

## RESULTS

Of 1,048 women screened, mean age was 40 years [interquartile range=34-46 years], 66% had a previous Pap test, 86% reported one lifetime sexual partner, and 91% were currently married (data not shown [3]). Of 192 women with abnormal cytology and/or HR-HPV-positivity who were referred for colposcopy, 159 (83%) attended (Table 1). In total, 36 CIN2+ and 23 CIN3+ cases (including 7 and 4 cases imputed among women without colposcopy) were included in the present analyses (Table 1). Only corrected indices are shown, but crude estimates can also be calculated from the data described in Table 1.

**Table 1:** CIN2+/3+ confirmed and imputed among 1,048 women aged ≥30 years, with and without colposcopy, respectively, by combination of cytology and HR-HPV results

Screening test results		Women with colposcopy			Women without colposcopy			All women				
Cytology	HR-HPV	N	Confirmed		N	Imputed^#^		N	CIN2+		CIN3+	
			CIN2+	CIN3+		CIN2+	CIN3+		n	%	n	%
Normal	-	0	0	0	856	0	0	856	0.0	0.0	0.0	0.0
ASCUS/AGUS/AGC	-	19	2	1	7	0.7	0.4	26	2.7	10.5	1.4	5.3
LSIL	-	6	1	0	2	0.3	0.0	8	1.3	16.7	0.0	0.0
ASC-H	-	9	0	0	0	0.0	0.0	9	0.0	0.0	0.0	0.0
HSIL+	-	2	1	0	0	0.0	0.0	2	1.0	50.0	0.0	0.0
Normal	+	94	11	5	19	2.2	1.0	113	13.2	11.7	6.0	5.3
ASCUS/AGUS/AGC	+	12	4	4	2	0.7	0.7	14	4.7	33.3	4.7	33.3
LSIL	+	8	2	2	0	0.0	0.0	8	2.0	25.0	2.0	25.0
ASC-H	+	5	4	4	1	0.8	0.8	6	4.8	80.0	4.8	80.0
HSIL+	+	4	4	3	2	2.0	1.5	6	6.0	100.0	4.5	75.0
**Total**		159	29	19	889	7	4	1,048	36	3.4	23	2.2

^#^For description of imputation see methods section. ASC-H=atypical squamous cells-cannot exclude high-grade squamous intraepithelial lesions; AGC=atypical glandular cells; AGUS=atypical glandular cells of undetermined significance; ASCUS=atypical squamous cells of undetermined significance; CIN2+=cervical intraepithelial neoplasia grade 2 or worse; CIN3+=cervical intraepithelial neoplasia grade 3 or worse; HR-HPV=high-risk human papillomavirus; HSIL=high-grade squamous intraepithelial lesions; LSIL=low-grade squamous intraepithelial lesions.

Table 2 shows screening indices of cross-sectional accuracy by different primary screening and triage methods. For primary cytology, screening test positivity was 7.5% for ASCUS+, and 5.1% for ASCUS+ with HR-HPV triage of ASCUS. For primary HR-HPV, positivity was 14.0% for HR-HPV and 3.2% for HR-HPV triage by ASCUS+ (Table 2). Test positivity for primary cytology at the threshold of LSIL+ was 3.7% (data not shown).

**Table 2:** Screening test accuracy to detect 36 CIN2+ and 23 CIN3+ among 1,048 women, after correction for missing biopsies

Primary screening		Triage test	Test positivity (%)^1^	Sensitivity (95%-CI)		Specificity (95%-CI)		PPV (95%-CI)		NPV (95%-CI)	
Test	Threshold			CIN2+	CIN3+	CIN2+	CIN3+	CIN2+	CIN3+	CIN2+	CIN3+
Cytology	ASCUS+	none	7.5	61* (44-77)	74^#^ (52-90)	94 (93-96)	94 (92-95)	28 (18-39)	22 (13-32)	98.6 (97.6-99.2)	99.4 (98.7-99.8)
		HR-HPV of ASCUS	5.1	56 (38-72)	70 (47-87)	97 (96-98)	96 (95-98)	38 (25-52)	30 (18-44)	98.4 (97.4-99.1)	99.3 (98.6-99.7)
GP5+/6+ PCR	HR-HPV	none	14.0	86* (70-95)	96^#^ (78-99)	88 (86-90)	88 (86-90)	21 (15-29)	15 (10-22)	99.5 (98.7-99.8)	99.9 (99.4-100.0)
		ASCUS+	3.2	47 (30-64)	70 (47-87)	98 (97-99)	98 (97-99)	50 (32-68)	47 (30-65)	98.1 (97.1-98.9)	99.3 (98.6-99.7)

* Relative sensitivity of HR-HPV versus ASCUS+ for CIN2+ = 1.41 (95% CI= 1.05 - 1.89)

^#^ Relative sensitivity of HR-HPV versus ASCUS+ for CIN3+ = 1.29 (95% CI= 1.00 - 1.67)

^1^ All percentages are based on the same denominator of 1,048 women.

ASCUS=atypical squamous cells of undetermined significance; CI=confidence interval; CIN2+=cervical intraepithelial neoplasia grade 2 or worse;

CIN3+=cervical intraepithelial neoplasia grade 3 or worse; HR-HPV=high-risk human papillomavirus; NPV=negative predictive value; PCR=polymerase chain reaction; PPV=positive predictive value.

HR-HPV testing showed higher sensitivity (86% and 96% for CIN2+ and CIN3+ respectively) than cytology ASCUS+ (61% and 74%), equating to sensitivity ratios of 1.41 and 1.29, respectively. Negative predictive value was also higher for HR-HPV (99.5% and 99.9% for CIN2+ and CIN3+, respectively) than cytology ASCUS+ (98.6% and 99.4%). Specificity, however, was lower for HR-HPV (88% for both CIN2+ and CIN3+) than for cytology ASCUS+ (94% for both), as was positive predictive value. Of note, increasing the cytology threshold to LSIL+ was associated with sensitivity of only 42% and 48% for CIN2+ and CIN3+, respectively (data not shown).

With respect to triage of primary cytology, triage of ASCUS by HR-HPV was associated with an improvement in specificity to 97% for CIN2+ and 96% for CIN3+, and improved positive predictive value (PPV), but with a decrease of sensitivity to 56% and 70%. Triage of primary HR-HPV by ASCUS+ was also associated with an improvement in specificity to 98% for both CIN2+ and CIN3+, and higher PPV, but also with a decrease of sensitivity to 47% for CIN2+ and 70% for CIN3+.

**Figure 1:**
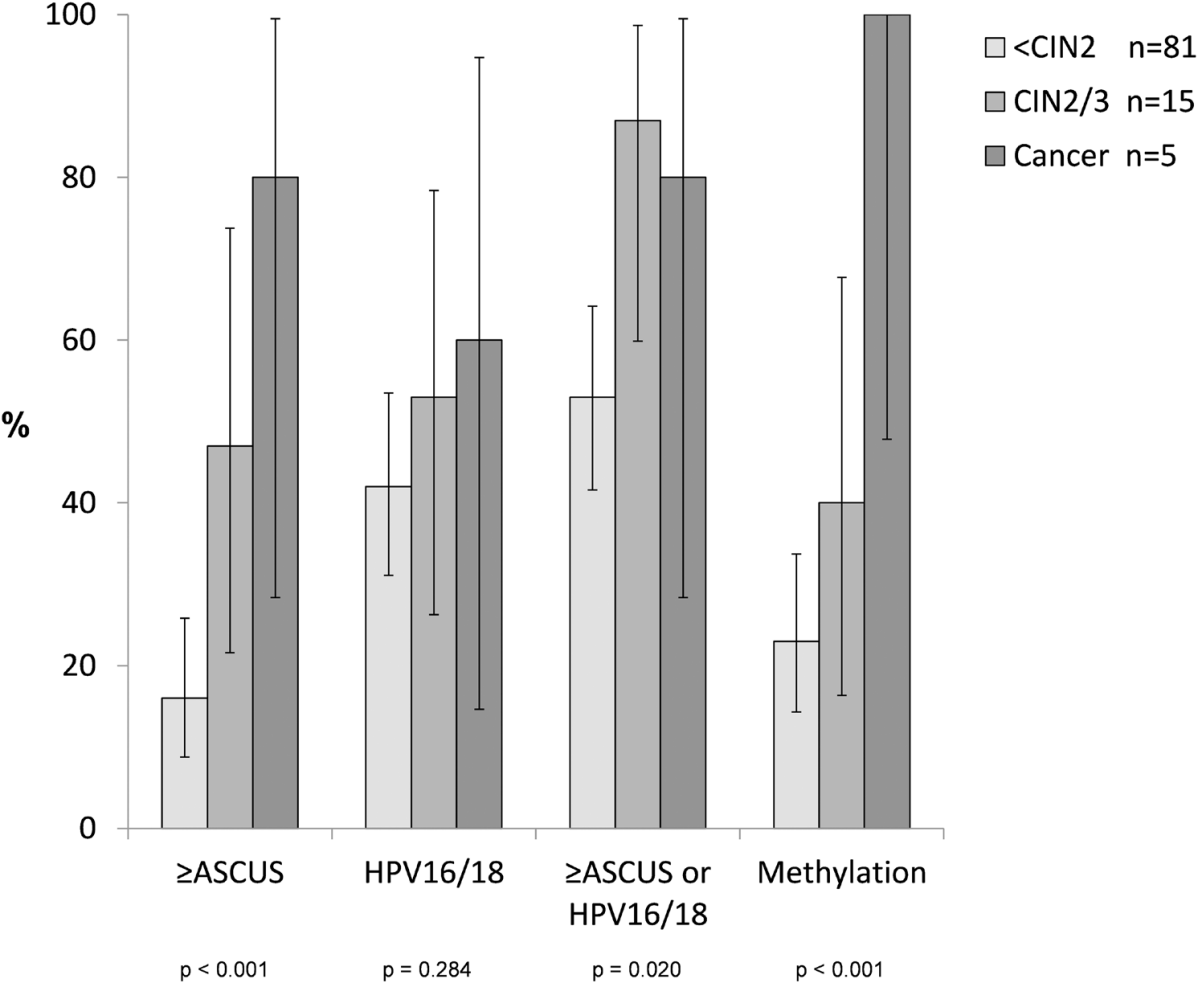
Test positivity1 for ASCUS+ cytology, HPV16/18 positivity, their contribution, and CADM1/MAL/miR124-2 methylation by disease grade among 101 HR-HPV positive women with colposcopy and biopsy ^1^ Error bars represent 95% confidence intervals of test positivity based on a binomial distribution. ASCUS=atypical squamous cells of undetermined significance; CIN=cervical intraepithelial neoplasia; HR-HPV=high-risk human papillomavirus.

To evaluate the potential utility of molecular markers for the triage of HR-HPV-positive women, we compared test positivity by histological diagnosis of HPV16/18 genotyping (alone or in combination with cytology) and CADM1/MAL/miR124-2 methylation, with that of ASCUS+ cytology, among a subset of 101 HR-HPV positive women (after exclusion of 19 without colposcopy/biopsy and an additional 22 without a valid result for CADM1/MAL/miR124-2 methylation) (Figure 1). The positivity of all three tests increased from <CIN2 (n=81), through CIN2/3 (n=15), to cancer (n=5). The trend in positivity was significant for ASCUS+ cytology (p<0.001) and CADM1/MAL/miR124-2 methylation (p<0.001), but not for HPV16/18 (p=0.284). Of note, all 5 cancers were CADM1/MAL/miR124-2 methylation positive. Combined ASCUS+ and/or HPV16/18 positivity was associated with lower discrimination across lesion grade in comparison to ASCUS+ alone or methylation, but offered the highest positivity in CIN2/3 (Figure 1).

## DISCUSSION

In this first evaluation of the cross-sectional performance of the cytology screening program in Bhutan, sensitivity of cytology ASCUS+ for CIN2+ (61%) and CIN3+ (74%) fell in the mid-range of estimates from similar studies that included colposcopy of HR-HPV-positive women, irrespective of whether they be in LMICs (26-87% for both CIN2+ and CIN3+) [7–15] or HICs (19-77% and 23-76%, respectively) [26, 27]. Specificity of ASCUS+ cytology in Bhutan (94% for CIN2+ and CIN3+) also fell in the mid-range of previous estimates (87-99% for both CIN2+ and CIN3+) [7, 9–15, 26–28].

Use of HR-HPV as a primary screening test was associated with a higher detection of CIN2+ and CIN3+ than cytology (sensitivity ratio = 1.41 and 1.29 respectively), consistent with findings from previous studies [7, 9–11, 13–15, 26–28], and a meta-analysis [29]. Higher detection rates of CIN2+ and CIN3+ have also been seen in the HR-HPV versus cytology arm of 8 randomized controls trials [29].

Performance of HR-HPV testing for detection of CIN2+ and CIN3+ has been shown to be heterogeneous across studies in LMICs [29]. In the present study from Bhutan, but for which HR-HPV testing was performed in a specialized laboratory in Europe, sensitivity of HR-HPV (96% for CIN3+) was towards the high end of estimates from previous reports (average 84%) in LMICs, reaching levels similar to that reported in HICs (average 98%) [29].

HR-HPV testing offered a higher cross-sectional negative predictive value than ASCUS+ cytology in Bhutan. Indeed, a negative HR-HPV test has also been shown to offer greater reassurance against future CIN3+ [30–33] and cervical cancer [31] in large prospective studies. Large randomized trials have also shown that primary HR-HPV screening results in a significantly lower incidence of CIN3+ [19] and cancer [34] than primary cytology. These data have led certain HICs to switch from cytology to HR-HPV as the primary screening test, including Australia, Italy, New Zealand, the Netherlands and the UK. World Health Organization [35] and U.S. guidelines [20] also recommend HR-HPV as a primary screening test.

Nevertheless, HR-HPV also resulted in a higher burden of referral to colposcopy and reduced specificity compared to cytology screening, consistent with results from previous cross-sectional studies [29]. Whilst some screening programs in LMICs have pragmatically chosen to treat all HR-HPV positive women on account of concerns of the accuracy/feasibility of triage options and of losses to follow up [36, 37], triage of HR-HPV positive women would be desirable to immediately refer only those at highest risk. The current recommended methods for triage in HICs include cytology ASCUS+ [18] or cytology in combination with HPV16/18 genotyping [19, 20], but host cell DNA methylation analysis is also a promising candidate [21–23], especially as it can also be performed on self-collected cervicovaginal samples [23].

In Bhutan, primary HR-HPV testing followed by ASCUS+ triage of HR-HPV-positives was associated with high specificity and PPV, requiring referral of only 3.2% of screened women (versus 14% for all HR HPV-positives), offering an option for triage in Bhutan, where cytology is already established. Although the sensitivity of cytology triage might have been higher if cytotechnicians had known that they were triaging HR-HPV-positive women, cytology triage in Bhutan was nevertheless associated with a substantial loss of cross-sectional sensitivity, missing more than one third of CIN2+. So in a subset of HR-HPV-positive women, we compared the discriminating power of two molecular-based triage options, namely HPV16/18 genotyping and host DNA methylation, to that of cytology. Although sample sizes were limited, CADM1/MAL/miR124-2 methylation was strongly related to the severity of cervical disease and was always positive in cervical cancer, as shown previously [38]. Indeed, methylation analysis appeared to offer similar discrimination of CIN2+ as ASCUS+ cytology, and better discrimination than HPV16/18 genotyping alone or in combination with cytology. Our findings are in agreement with large recent studies that noted that DNA methylation analysis (although not always of the same combination of gene markers) was a non-inferior triage option versus cytology, in both clinician-collected [21, 22] and self-collected [23] HR-HPV-positive samples, and actually performed significantly better than HPV16/18 genotyping [21, 22].

Prior to becoming a recommended primary screening test, HR-HPV testing was recommended to triage ASCUS in primary cytology programs, being shown to have higher sensitivity and similar specificity than repeat cytology in this group of women [39]. However, this algorithm was associated with relatively poor sensitivity in Bhutan (56% for CIN2+), substantially lower than in a larger Chinese study (84%) [40].

The strengths of this study were the high proportion of screen-positive women who received colposcopy and biopsy, and the fact that histology was imputed among the few who did not (although correction had little effect on sensitivity and specificity estimates). Furthermore, the population-based sample is expected to be broadly representative of women aged ≥30 years living in Bhutan, and the risk of HR-HPV-positivity and CIN3+ in this population was high, as reported previously [24]. The major limitations were the restricted sample size and the fact that HR-HPV testing, HPV16/18 genotyping and methylation analysis was not performed locally, but in an expert laboratory in Europe, so that the performance of the assays did not truly represent that of field conditions. Nevertheless, the clinical performance of the GP5+/6+ polymerase chain reaction (PCR)-based assay in Amsterdam has been shown to be almost identical to that of the more widely used HC2 [relative CIN2+ sensitivity=1.00 (0.96-1.04) and specificity=0.99 (0.91-1.07)] [25, 41]. Lastly, we are aware that a prospective evaluation of screening algorithms involving repeat screening rounds may lead to different relative performance of HR-HPV versus cytology, but this is also dependent on a country's willingness and capacity to implement organized follow-up.

In conclusion, despite the relatively good performance of the Bhutanese cytology program, a shift to primary HR-HPV screening has the potential to further improve detection of cervical pre-cancer, albeit with a higher referral rate for colposcopy and loss of specificity. Cytological triage of HR-HPV-positive women diminishes immediate referral to colposcopy but would have missed more than one third of CIN2+. Whilst methylation analysis was shown to be a promising and objective alternative to ASCUS+ cytology in the triage of HPV-positive women samples, our results remain preliminary.

## MATERIALS AND METHODS

### Population

The study had the approval of both the Research Ethical Board of the Bhutan Ministry of Health and the IARC Ethics Committee.

In 2012, during a population-based survey of HPV prevalence, 2,505 women aged 18-69 years were invited and underwent a gynecological examination in Jigme Dorji Wangchuck National Referral Hospital (JDWNRH) and Lungthenphu Hospital, Thimphu, Bhutan. Study procedures have been described in detail elsewhere [24]. Exfoliated cervical cells were obtained using a cytobrush (Rovers Medical Devices, The Netherlands). After preparation of a conventional Pap smear, the brush containing cellular material was placed in a vial containing PreservCyt medium for HPV and methylation testing.

The present study is restricted to the subset of these women among whom HR-HPV screening is recommended, namely 1,048 women aged ≥30 years. As Pap smear, and later HR-HPV, results became available, first all women with abnormal cytology (N=79), and subsequently, also HR-HPV-positive women with normal cytology (N=113), were referred for colposcopy, of whom 83% finally attended (159 of 192) (Table 1).

### Cervical disease assessment

Colposcopy was used to obtain biopsies from all suspicious areas among women with abnormal colposcopical findings. Cervical biopsies were obtained from 105 (66%) of 159 women who underwent colposcopy. Histology was performed at JDWNRH, Thimphu, and 29 cases were diagnosed as CIN2+ (including 10 CIN2, 14 CIN3 and 5 cervical cancers) (Table 1). Treatment of colposcopy-detected lesions was performed according to local protocols, primarily using loop electrosurgical excision procedures for CIN2/3.

### HPV testing and genotyping

Vials containing cellular material in PreservCyt medium were shipped to the Department of Pathology at the VU University Medical Center, Amsterdam. DNA was first extracted from the PreservCyt sample using magnetic beads on a robotic system. β-globin PCR analysis was then conducted to confirm the presence of human DNA in all specimens [42] and a general primer GP5+/6+ -mediated PCR was used to amplify HPV DNA. HPV positivity was assessed by hybridization of PCR products in an enzyme immunoassay with two oligoprobe cocktails that, together, can detect 44 mucosal HPV types. Subsequent HPV genotyping was conducted by reverse-line blot (RLB) hybridization of GP5+/6+ PCR products as described previously [42, 43]. HR-HPV refers to positivity for 13 high-risk HPV types only (16, 18, 31, 33, 35, 39, 45, 51, 52, 56, 58, 59 and 68) [44]. HPV16/18 genotyping refers to positivity for HPV16 and/or HPV18. Non-high-risk HPV types detected by GP5+/6+RLB are ignored.

### Host DNA methylation analysis

CADM1/MAL/miR124-2 methylation analysis was performed at the Department of Pathology at the VU University Medical Center, Amsterdam, as previously described [38]. In brief, extracted DNA was first subjected to bisulfite treatment using the EZ DNA Methylation Kit (Zymo Research, USA). DNA methylation analysis was performed by a commercial multiplex quantitative methylation-specific PCR (PreCursor-M) which enables simultaneous amplification and detection of methylated DNA of CADM1, MAL and miR-124-2, and methylation-independent β-actin as sample quality control, within a single reaction [45]. This combination of three genes was chosen based upon prior optimization and validation work on cervical samples [38]. Samples were scored methylation positive for CADM1, MAL and miR-124-2 relative to that of β-actin, according to manufacturers’ instructions (based on validated thresholds that on a validation set of cervical scrapes of HR-HPV-positive women gave rise to a maximum CIN3+ sensitivity at 70% specificity), as described previously. A sample was considered positive if any of the three genes scored positive.

### Statistical analysis

Cytology and HR-HPV testing were first compared as stand-alone primary screening tests. In addition, different triage approaches for immediate referral of women to colposcopy were evaluated. These included two well-established protocols (HR-HPV testing of ASCUS cytology only, and ASCUS+ cytology of HR-HPV-positive women). Conventional screening indices of accuracy, including sensitivity, specificity, PPV, negative predictive value and their 95% confidence intervals were calculated for both CIN2+ and CIN3+. Firstly, crude indices using only CIN2+ and CIN3+ histologically confirmed among the 159 women attending colposcopy were calculated, assuming that all women without a biopsy were histologically negative. Secondly, corrected indices were calculated after imputation of missing data for the 33 HR-HPV-positive and/or cytologically abnormal women who did not attend colposcopy [7, 40]. In the corrected model, observations were replaced by pseudo observations weighted by the probability of CIN2+/3+ among women with the same combination of HR-HPV and cytology results, and who underwent colposcopy. Lastly, in order to evaluate their potential to triage HR-HPV-positive women, we also compared ASCUS+ cytology, HPV16/18 genotyping, their combination, and host DNA methylation analysis across cervical diagnosis severity, among a subgroup of 101 HR-HPV-positive women (namely those who underwent colposcopy/biopsy and had a valid result for all three tests), using a chi-squared test for trend across grades of <CIN2, CIN2/3 and cancer.

## Abbreviations

ASCUS: atypical squamous cells of undetermined significance
CI: confidence interval
CIN: cervical intraepithelial neoplasia
HR-HPV: high-risk human papillomavirus
HSIL: high-grade squamous intraepithelial lesions
JDWNRH: Jigme Dorji Wangchuck National Referral Hospital
LMIC: low- and middle-income country
LSIL: low-grade squamous intraepithelial lesions
Pap: Papanicolaou smears
PR: prevalence ratio
